# Anesthetic management of tracheal stent extraction using a double gum elastic bougie technique

**DOI:** 10.1186/s40981-022-00500-z

**Published:** 2022-02-03

**Authors:** Satoshi Sato, Tomohiro Chaki, Takayuki Onaka, Michiaki Yamakage

**Affiliations:** Department of Anesthesiology, Sapporo Medical University School of Medicine, South 1, West 16, Chuo-ku, Sapporo, Hokkaido 060-8543 Japan

**Keywords:** Tracheal stenosis, Self-expandable metallic stent, Airway obstruction, Tracheostomy

## Abstract

**Background:**

Tracheal stenosis is a life-threatening condition, and management of a patient with a risk of tracheal stenosis is challenging for anesthesiologists. In this report, we describe a method for airway management using two gum elastic bougie method when removing a tracheal stent via a tracheostomy orifice with a risk of airway restenosis.

**Case presentation:**

A 71-year-old man had an enlarged squamous cell carcinoma of the lung invading the upper mediastinum that had caused severe stenosis of the trachea. Two months after diagnosis, a tracheal stent had been placed to maintain tracheal patency. One month after stent placement, acute respiratory failure was induced by upper airway obstruction caused by subglottic airway edema due to mechanical stimulation of the cranial end of the stent, and the patient was rescued by oral tracheal intubation. Tracheal stent extraction was scheduled to relieve the laryngeal edema. Since there was a risk of tracheal restenosis because of the possibility of accidental evulsion of the orally tracheal tube which intubated to secure an emergency airway and tracheal stent extraction, two gum elastic bougies were inserted through the oral tracheal tube and tracheostomy orifice to facilitate re-intubation. After extraction of the tracheal stent, airway openness was maintained and tracheostomy was completed without any complication.

**Conclusion:**

Successful management of tracheal stent extraction was performed using a double gum elastic bougie technique.

## Background

Tracheal stent placement is indicated for patients who have airway stenosis of 50% or more and a risk of asphyxia [1]. Tracheal stenting is considered to be the most rapid and effective way for relieving tracheal stenosis and improving respiratory status, and it has been used as a bridge therapy to relieve symptoms and buy time before subsequent treatment [2]. Hybrid stents, which have been increasingly used in recent years, have been designed and developed to combine the properties of both conventional silicone stents and metal stents. Hybrid stents are designed to prevent major complications, including granulation, tumor invasion, and breakage of the metal part, that have been reported with metal stents while maintaining the characteristics of existing silicone stents, which prevent tumor invasion from blocking the lumen, and the advantages of metal stents, which are easier to place. Hybrid stents are useful for patients in whom stent removal after additional treatment is planned [3, 4]. However, in the tracheal stent extraction procedure, careful management is needed to prevent tracheal restenosis and obstruction by accidental extubation and stent removal. Complications during stent removal include bleeding, laceration of the tracheal mucosa, restenosis requiring stent reimplantation, damage to the pulmonary artery, pneumothorax, and residual stent debris [5].

The safety and efficacy of oral tracheal tube replacement using a bougie to maintain airway integrity have been reported [6]. In recent years, the tracheostomy technique using a bougie has been used to ensure the safety of surgical emergency airway management in the field of emergency medicine [7]. However, there have been only a few reports on anesthetic management of a patient who had a tracheal stent removed via a tracheostomy orifice [8]. Here, we report our novel method for airway management by maintaining a route for oral and transtracheal reintubation using two bougie tubes during tracheal stent removal with a risk of restenosis.

## Case presentation

We obtained written informed consent from the patient for publication of this case report. The manuscript adheres to the CAse REport (CARE) guidelines.

The patient was a 71-year-old man who had a history of hypertension and had been smoking for more than 40 years. He complained of hoarseness and was diagnosed with poorly differentiated squamous cell carcinoma of the lung 2 months ago. The tumor was located on the right side of the upper mediastinum. One month earlier, the tumor was 40 mm in size and was compressing the trachea, causing severe tracheal stenosis to the extent that the inner diameter of the trachea was only 11 mm. To maintain tracheal patency, an AERO^TM^ stent (Merit Medical Systems, Salt Lake City, UT), which is a hybrid stent with full metal stent coverage and a length of 75 mm, was implanted from the cricoid cartilage to the cranial side of the tracheal bifurcation. One month after implantation, acute hypoxia associated with airway obstruction in the hospital ward, the patient presented with acute respiratory failure and was rescued by oral tracheal intubation. Remarkable laryngeal edema was observed at that time. After intubation, endotracheal edema from the subglottis to the cranial end of the stent was observed using bronchofibers. As a result of consultation with respiratory physicians, we made a diagnosis of acute airway obstruction caused by tracheal stent-induced subglottic edema. On the same day, tracheostomy and tracheal stent extraction through the tracheostomy orifice were scheduled to attenuate the airway edema. Two airway risks were considered preoperatively: the risk of airway restenosis due to accidental removal of the tracheal intubation tube and the risk of the trachea being restenotic after tracheal stent extraction due to bleeding from the tumor or release of pressure drainage. We planned for veno-venous extracorporeal membrane oxygenation (VV-ECMO) with blood flow set at 2 L•min^-1^ by femoral arteriovenous pumping and draining to be established before tracheostomy and tracheal stent extraction.

**Fig. 1:**
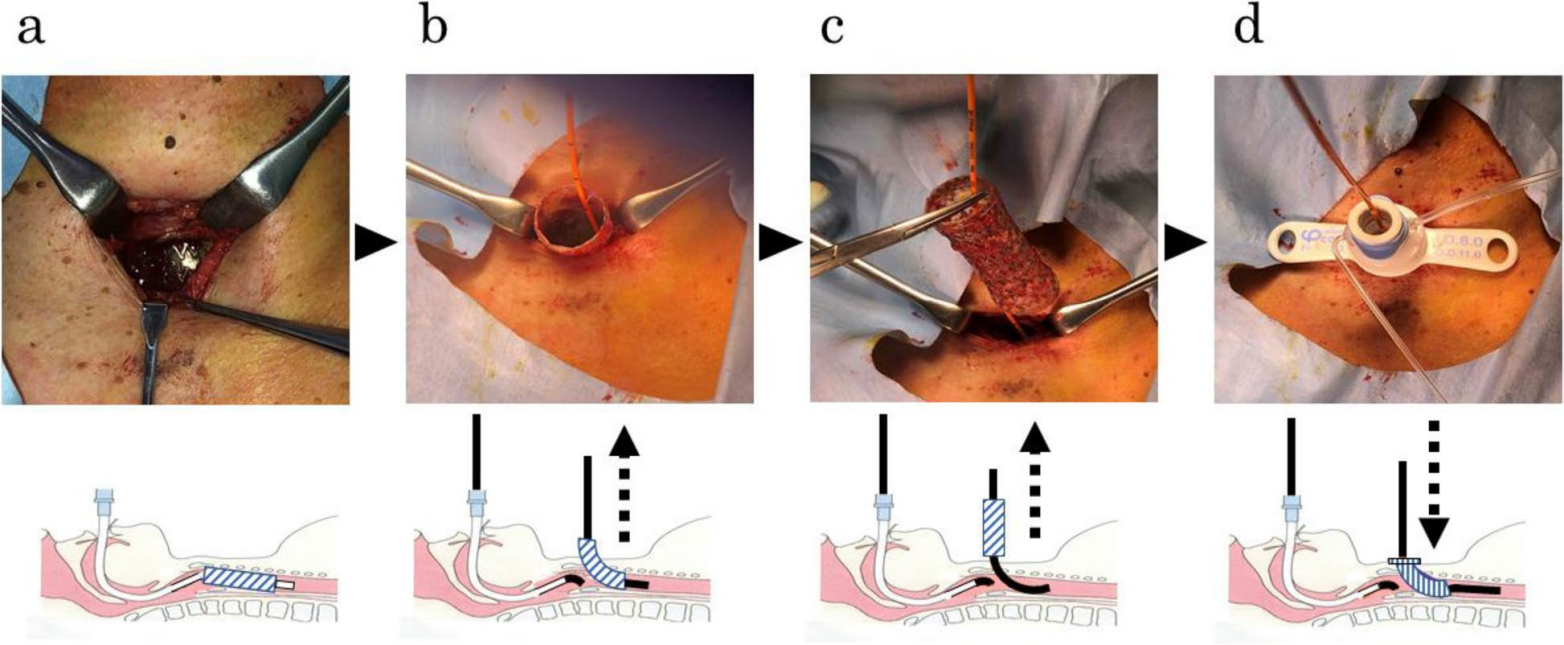
Schema of the double gum elastic bougie technique. **a** The tracheal stent was exposed when the tracheal window was opened at the inferior border of the cricoid cartilage. **b** A gum elastic bougie was passed into the orally intubated tube, and the intubation tube was pulled to enable extraction of the tracheal stent from the tracheostomy. The cranial end of the tracheal stent was pulled out through the tracheal opening window. Another gum elastic bougie was passed through the tracheal stent lumen and advanced to the tracheal bifurcation. **c** The tracheal stent was removed while maintaining a secure airway through the orally intubated tube and the tracheal opening using two gum elastic bougies. **d** After removal of the tracheal stent, a tracheostomy tube was inserted through the gum elastic bougie

**Fig. 2:**
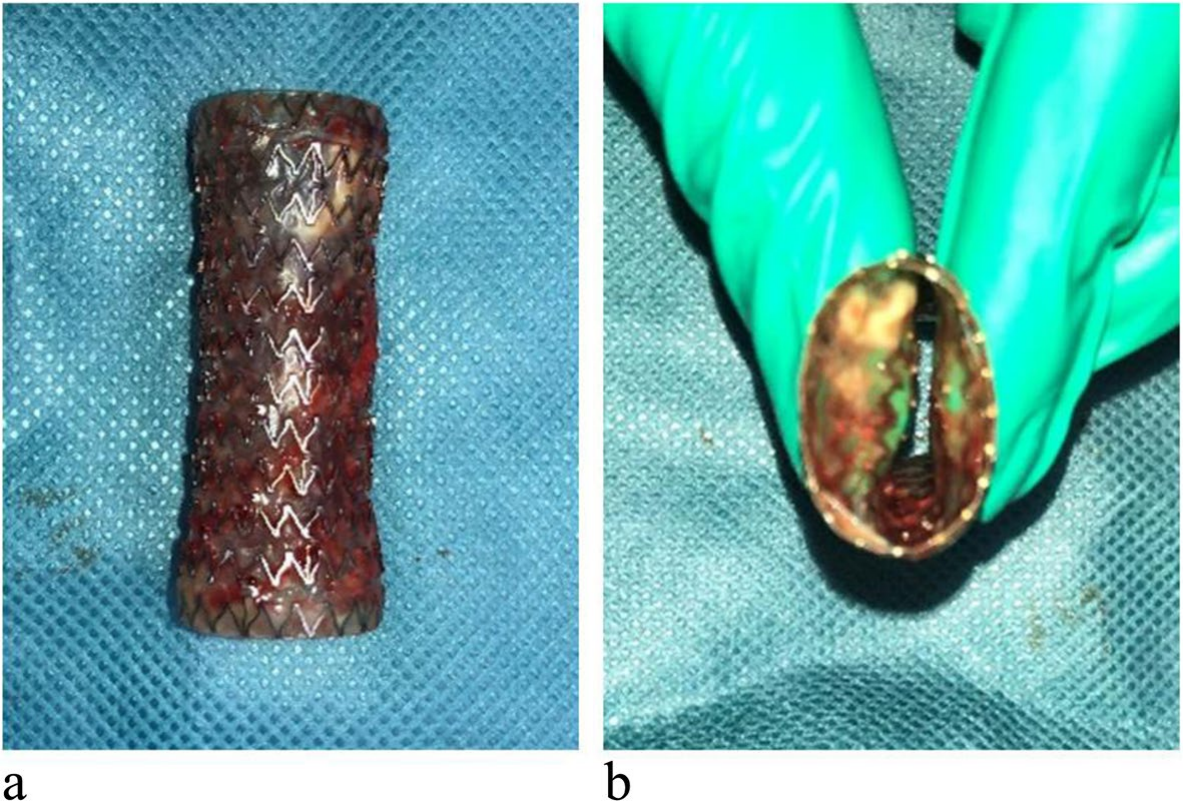
Removal of the AERO^TM^ stent in this case. **a** The AERO^TM^ stent is a full-coverage stent in which the entire wall of the metal stent is covered with a polyurethane membrane. Because the entire metal part is covered by the membrane, it does not become implanted into the airway and is easy to remove and modify. **b** The inside of the stent has a hydrophilic coating, which reduces fluid retention and is flexible enough to be removed

General anesthesia was induced with 40 mg propofol and 50 mg rocuronium and was maintained with 1.2% sevoflurane and 0.06 μg•kg^-1^•min^-1^ remifentanil infusion. After the establishment of VV-ECMO, blood flow was set at 2 L•min^-1^ and heparin was administered to maintain activated clotting time at about 200 s. An inverted U-shaped tracheal incision was first made at the inferior margin of the cricoid cartilage (Fig. 1a). Next, the tip of the oral tracheal intubation tube was moved to the oral side of the tracheal opening with a gum elastic bougie passed through the lumen of the tracheal intubation tube to facilitate re-intubation after accidental extubation. The cranial end of the tracheal stent was withdrawn from the tracheal opening. Another gum elastic bougie was advanced through the tracheal stent lumen to the caudal side of the tracheal stent (Fig. 1b). The tracheal stent was removed via the orifice of the tracheostomy by the surgeon using forceps to pinch and pull it out while maintaining the methods to secure the airway by the oral and trans-tracheal gum elastic bougies (Figs. 1c and 2). After removal of the tracheal stent, a tracheostomy tube (Adjust Fit®, Fuji Systems Corp., Tokyo, Japan) was inserted through the trans-tracheal gum elastic bougie (Fig. 1d). The reason for using two bougies was because the edema was aggravated from the subglottis to the cranial end of the stent, and we assumed that the trachea could be easily obstructed during the replacement operation, in which the oral tracheal tube was pulled up to the cranial side and the tracheal tube was inserted through the tracheostomy orifice. Appropriate oxygenation was maintained throughout the procedures. After completion of the tracheostomy, the patient was weaned from VV-ECMO and discharged to the intensive care unit with a ventilator. Four hours after the operation, the patient had the tracheostomy cannula removed, and his respiratory and circulatory status remained stable.

## Discussion

Airway management for tracheal stent extraction with the risk of re-occlusion through a tracheostomy orifice was performed safely using a double elastic bougies technique. The bougie-assisted cricothyrotomy method, in which an endotracheal tube is inserted using a gum elastic bougie as a guide, is used for surgical cricothyrotomy in emergency medicine to secure an emergency airway [7]. On the basis of the bougie-assisted cricothyrotomy, we developed a double gum elastic bougie technique for the airway management in this case. Since the double gum elastic bougie technique allows re-intubation orally or trans-tracheally by each bougie, this method might enable safe and constant maintenance of airway security.

In this case, a gum elastic bougie was placed through an oral intubation tube and a tracheostomy to maintain the airway during the procedures. In the case of acute airway obstruction associated with stent removal, the tracheal tube or tracheostomy tube may advance into the mediastinum or subcutaneously even if airway clearance is attempted through the tracheostomy orifice. At first, a gum elastic bougie was placed through the oral tracheal tube before starting the tracheal stent extraction. There were reasons for this bougie insertion. There was concern about the possibility of the orally inserted tracheal tube being accidentally removed before the tracheal orifice was established, and it was assumed that oral re-intubation would be difficult if drastic airway restenosis occurred due to emergency tracheal intubation-induced deterioration of the subglottic edema. The surgeon inserted the gum elastic bougie into the airway through the tracheostomy orifice and then removed the tracheal stent and could easily insert the tracheostomy tube. The bougie insertion via the tracheostomy orifice was necessary because there was a possibility of the trachea being obstructed by the tumor immediately after removal of the tracheal stent from the tracheostomy orifice. In that case, a tracheal tube had to be urgently inserted to secure the airway, and it served as a guidewire for this procedure. Our technique enabled maintenance of continuous airway management throughout the tracheal stent removal procedure.

Major complications of stents include lower respiratory tract infection, pneumonia, granulation, mucus retention and obstruction, hemoptysis, and stent deviation [9], with granulation reported in 0–20% of cases and migration and deviation reported in 10–22% of cases in which silicone stents were used [10]. In this case, it was considered that stent placement may cause edema at the end of the stent. The metal edge of the stent exerts circumferential pressure on the tissue and is likely to cause airway inflammation and subsequent granulation tissue formation [11]. Taking these factors into consideration, the subglottic space and the upper part of the trachea should be treated carefully because these areas are prone to granulation due to impaired mucosal blood flow caused by mechanical compression of the tracheal wall [12].

In this case, because of the appearance of laryngeal edema, it was difficult to remove the stent through the larynx. Thus, the tracheal stent had to be removed through a tracheostomy. It has been suggested that it is essential to maintain a width of at least 1 cm between the upper edge of the stent and the vocal folds to prevent discomfort and obstructive complications such as laryngeal edema, stent migration, and granulation tissue [13]. In this case, the distance between the superior edge of the stent and the vocal folds was about 1 cm, and the patient was at high risk of complications due to the stent.

Although there is no evidence regarding how general anesthesia should be managed and whether or not muscle relaxants should be used during stent placement [14], stent removal should be performed using a rigid bronchoscope under general anesthesia [15]. There have been reports of restenosis caused by granulation after stent removal, stent rupture during removal, and tracheal injury, although these occurred during metal stent removal. Careful treatment and airway management considering airway re-stenosis are therefore required [16–18]. In addition, stent extraction with rigid bronchoscopy is recommended to deal with complications during extraction [19]. The airway stimulation during stent extraction makes it difficult to maintain the depth of anesthesia, and management under spontaneous breathing is required in some cases. In this case, we chose sevoflurane to facilitate the adjustment of anesthesia depth. However, because adequate ventilation is a prerequisite for emergence from anesthesia with sevoflurane, anesthetic management with total intravenous anesthesia, which does not depend on ventilation, may be more appropriate.

## Conclusion

Using our newly developed double gum elastic bougie technique, it was possible to safely manage tracheostomy and tracheal stent extraction in a patient with a risk of airway restenosis.

## Data Availability

The authors declare that all data supporting the findings of this report are available within the article.

## Abbreviation

VV-ECMO: Veno-venous extracorporeal membrane oxygenation
